# Exploring the Genetic Roles of Diet and Other Modifiable Risk Factors in the Risk of Angina: A Causal Investigation Using Mendelian Randomization in UK Biobank and FinnGen Cohorts

**DOI:** 10.3390/life14070905

**Published:** 2024-07-20

**Authors:** Essam Al Ageeli

**Affiliations:** Department of Basic Medical Sciences (Medical Genetics), Faculty of Medicine, Jazan University, Jazan 45142, Saudi Arabia; ealageeli@jazanu.edu.sa

**Keywords:** angina pectoris, mendelian randomization, diet, smoking, body mass index, physical activity, UK Biobank, FinnGen

## Abstract

Background: Angina pectoris, a debilitating manifestation of coronary artery disease, has been associated with various modifiable risk factors. However, the causal underpinnings of these associations remain unclear. This study leveraged Mendelian randomization (MR) to investigate the causal roles of dietary patterns, smoking behaviors, body mass index (BMI), and physical activity in the development of angina. Methods: Two-sample MR analyses were performed using summary-level data from large-scale genome-wide association studies (GWASs) and biobank resources, including the UK Biobank (UKB) and FinnGen cohorts. Genetic variants associated with various types of exposure such as fruit and salad intake, smoking initiation and intensity, BMI, and physical activity were used as instrumental variables, and their causal effects on angina risk were assessed. Results: In the UKB cohort (336,683 individuals, 10,618 cases), genetically proxied fruit (OR = 0.95, 95% CI: 0.93–0.97) and cheese intake (OR = 0.98, 95% CI: 0.97–0.99) were associated with decreased angina risk, while smoking initiation (OR = 1.01, 95% CI: 1.002–1.012), maternal smoking (OR = 1.06, 95% CI: 1.03–1.09), and BMI (OR = 1.01, 95% CI: 1.01–1.02) were associated with increased risk. In the FinnGen cohort (206,008 individuals, 18,168 cases), fruit (OR = 0.30, 95% CI: 0.17–0.53) and salad intake (OR = 0.31, 95% CI: 0.12–0.55) were found to be protective, while smoking initiation (OR = 1.20, 95% CI: 1.04–1.37) and intensity (OR = 1.15, 95% CI: 1.04–1.26) and BMI (OR = 1.31, 95% CI: 1.18–1.47) increased angina risk. Conclusions: This study provides robust evidence for the causal roles of various modifiable risk factors associated with angina development, highlighting the potential benefits of dietary interventions that promote increased fruit and vegetable consumption, smoking cessation, and weight management to mitigate angina risk. Further investigation is needed to generalize these findings to populations with diverse genetic backgrounds, lifestyles, and environmental exposures.

## 1. Introduction

Angina pectoris, a prominent manifestation of coronary artery disease (CAD), affects millions of people worldwide and imposes substantial economic and public health burdens [1]. While traditionally attributed to atherosclerotic plaque accumulation, recent evidence underscores the multifactorial etiology of angina, implicating modifiable risk factors such as diet, smoking, and physical inactivity in its development [2,3]. Unraveling the causal underpinnings of these associations is crucial for informing targeted preventive strategies and mitigating the impact of this pervasive condition.

Conventional observational studies have linked various dietary and lifestyle factors to angina risk; however, their interpretability is often hindered by residual confounding and reverse causation bias [4]. Mendelian randomization (MR), a novel analytical approach that leverages genetic variants as instrumental variables, offers a powerful framework to probe putative causal relationships while circumventing these limitations [5]. By exploiting the random assortment of genetic variants during gamete formation, MR mimics the principles of randomized controlled trials, enabling causal inference from observational data [6].

While conventional observational studies have provided valuable insights, they are often limited by confounding factors and reverse causation. Mendelian randomization (MR) addresses these limitations by using genetic variants as proxies for exposures, mitigating issues of confounding and providing insights into lifelong exposures [7]. This is particularly relevant for chronic conditions like angina that develop over extended periods. Mendelian randomization (MR) uses genetic variants as proxies for modifiable exposures to infer causal relationships, overcoming limitations of traditional observational studies such as confounding and reverse causation [8]. In our study, MR leverages genetic variants associated with exposures (e.g., dietary patterns, smoking behaviors) to estimate their causal effects on angina risk. This approach is particularly valuable for studying chronic conditions like angina, where randomized controlled trials may be impractical or unethical. By examining the lifelong effects of various risk factors, MR provides robust evidence that can inform targeted preventive strategies and personalized interventions.

The present study leverages the strengths of MR and large-scale genetic data from the UK Biobank and FinnGen cohorts to investigate the causal relationships between various modifiable risk factors and angina. This approach not only addresses the limitations of previous observational studies but also offers the potential to identify novel targets for prevention and intervention strategies.

The current study harnesses the strengths of MR to elucidate the causal roles of various modifiable risk factors, including dietary patterns, smoking behaviors, body mass index (BMI), and physical activity, in the development of angina. By integrating summary-level data from large-scale genome-wide association studies (GWASs) and biobank resources, we aim to provide robust evidence to guide public health interventions and inform clinical decision making regarding this prevalent cardiovascular condition.

Importantly, this investigation leverages data from two distinct population-based cohorts: the UK Biobank (UKB) and FinnGen. The UKB, a prospective study of over 500,000 individuals recruited from across the UK between 2006 and 2010, has emerged as a valuable resource for genetic and epidemiological research [9]. Complementing this resource is FinnGen, a comprehensive Finnish genetic research initiative aiming to enroll 500,000 participants by 2025, that offers insights into a genetically isolated population [10]. Using these large-scale datasets, our study seeks to investigate the causal relationship between diet and other modifiable factors and the risk of angina, paving the way for targeted preventive measures and personalized treatment strategies for angina. Nevertheless, it is crucial to acknowledge that the generalizability of these findings to populations with different genetic backgrounds, lifestyles, and environmental exposures may be limited, given that the UKB and FinnGen cohorts are predominantly composed of individuals of European ancestry.

The primary objective of this study is to use Mendelian randomization to investigate the causal relationships between modifiable risk factors (including dietary patterns, smoking behaviors, BMI, and physical activity) and the development of angina. By leveraging genetic data from large-scale cohorts, I aim to provide robust evidence for the causal roles of these factors in angina risk, potentially informing targeted preventive strategies and personalized interventions.

## 2. Material and Methods

The current study utilized two-sample MR to investigate the causal relationships between the study variables and the risk of angina.

**Figure 1:**
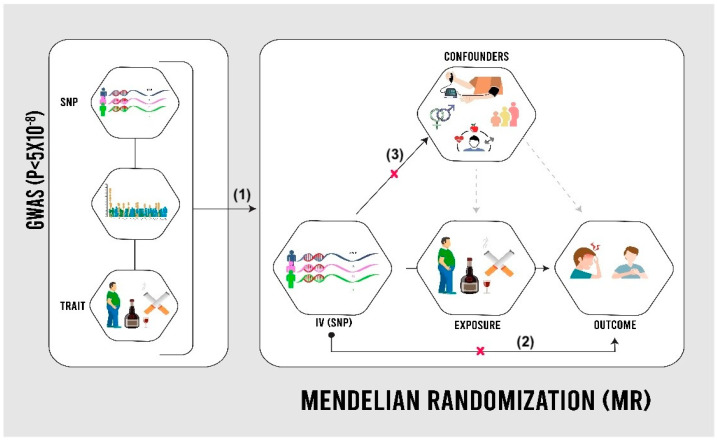
Mendelian randomization (MR) design [13]. SNPs: single-nucleotide polymorphisms. As shown in the image, the validity of the Mendelian randomization approach relies on three assumptions: (1) genetic variants used as instruments are strongly associated with the exposure, (2) these variants affect the outcome only through the exposure (exclusion restriction), and (3) the genetic instruments are not linked to confounding factors that influence both the exposure and outcome (independence).

MR utilizes genetic variations in the human genome, known as single-nucleotide polymorphisms (SNPs), to establish causal relationships between modifiable risk factors and outcomes [5]. GWASs have identified SNPs associated with various traits, such as smoking behavior and BMI, using a significance threshold of *p* < 5 × 10^−8^ [11]. In MR analysis, SNPs act as proxies for risk factors, overcoming the limitations of observational data, such as confounding and reverse causation (Figure 1). Assumptions include significant SNP–risk factor associations, the lack of a direct SNP–outcome relationship, and the absence of confounding [12]. The study utilized publicly available summary-level data from the UKB, a cohort of 502,000 individuals assessed between 2006 and 2010 across the UK, and FinnGen, a Finnish genetic research initiative aiming for 500,000 participants by 2025 [9,10]. In both cohorts, angina cases were identified using ICD codes from health records. Individuals with pre-existing cardiovascular disease at baseline were excluded.

This study employed a two-sample Mendelian randomization (MR) approach to investigate causal relationships between various exposures and angina risk. MR uses genetic variants as instrumental variables to estimate causal effects, leveraging summary-level data from genome-wide association studies (GWAS) for both exposures and outcomes in the UK Biobank and FinnGen cohorts. The validity of MR analysis relies on three key assumptions: strong association between genetic variants and exposure, absence of association between variants and confounders, and variants affecting the outcome only through the exposure. To address these assumptions, this study selected genetic instruments with genome-wide significance (*p* < 5 × 10^−8^), conducted sensitivity analyses using MR–Egger regression to detect pleiotropy, used multiple genetic variants when possible, and compared results across different MR methods. While these strategies strengthen the reliability of results, potential residual violations of assumptions are considered in the interpretation of findings.

The current investigation examined the potential causal roles of diet, smoking habits, BMI, and physical activity in angina development. Relevant variables were identified by leveraging genome-wide significant SNPs from Pirastu et al. [14], the UKB [15], GWAS and the Sequencing Consortium of Alcohol and Nicotine use (GSCAN) [16], the Genetic Investigation of Anthropometric Traits (GIANT) [17], and Klimentidis et al. [18]. The included SNPs represent genetic variations associated with specific traits, as determined through GWASs at a significance threshold of *p* < 5 × 10^−8^ [12]. The selection criteria for SNPs as genetic instruments were based on their genome-wide significance (*p* < 5 × 10^−8^) in previous GWAS studies, ensuring a strong association with the exposure of interest. This threshold was chosen to minimize the risk of weak instruments. The use of multiple SNPs for each exposure enhances the precision of the MR estimates. While the robustness of individual SNPs could not be directly investigated in this two-sample MR design, all included SNPs met the assumptions of MR based on their original studies. Additionally, SNPs were pruned for linkage disequilibrium to ensure independence, and those with known pleiotropic effects were excluded to maintain the validity of the MR assumptions.

The study utilized a specific group of SNPs that have been widely referenced in the literature [19,20,21] for each exposure: 41 that act as a proxy for fruit intake [14], 93 for smoking initiation [8], 23 for smoking intensity [15], 7 for maternal smoking [22], 22 for salad intake [23], 79 for BMI [24], and 11 for physical activity [18]. After harmonizing the genetic data related to angina (i.e., aligning and standardizing the reporting of genetic associations to ensure the consistent expression of effect alleles for exposures and outcomes with each additional copy of the same allele), we examined a set of SNPs for each exposure factor in relation to angina.

MR and sensitivity analyses were conducted using the *TwoSampleMR* package in R software (version 4.2.3) R Development Core Team, Vienna, Austria). Initially, genetic data for exposures and their respective outcomes were retrieved. Subsequently, MR analyses for angina were performed using data from both the UKB and FinnGen consortia. A significance threshold of *p* < 0.05 was applied to all MR analyses with a primary focus on the inverse variance weighted (IVW) method. Additionally, more stringent MR measures, such as MR–Egger, were employed to detect any potential deviations from IVW results, particularly considering the possibility of increased pleiotropy [11].

## 3. Results

### 3.1. Genetic Profiling of Risk Factors

The MR analysis relies on three key assumptions: strong SNP–exposure association, no SNP–confounder association, and SNP influence on the outcome only through the exposure. These were assessed by ensuring the genome-wide significance of SNPs (*p* < 5 × 10^−8^), examining SNP–confounder associations using GWAS data, and employing MR–Egger regression to detect pleiotropy. Multiple sensitivity analyses, including weighted median and mode-based estimates, were used to assess result robustness across different MR methods.

A total of 353 SNPs covering multiple risk factors were assessed with each factor examined through a spectrum of 11 to 93 SNP variants. These genetic markers were sourced from diverse consortia with varying sample sizes ranging from 64,949 to 632,802 individuals per risk factor (Table 1).

### 3.2. Genetic Characteristics Associated with Angina

Angina risk was examined in two cohorts: the UKB (336,683 individuals; 10,618 cases, 326,065 controls) and FinnGen (206,008 individuals; 18,168 cases, 187,840 controls).

#### 3.2.1. Angina Risk in the UKB Cohort

**Figure 2:**
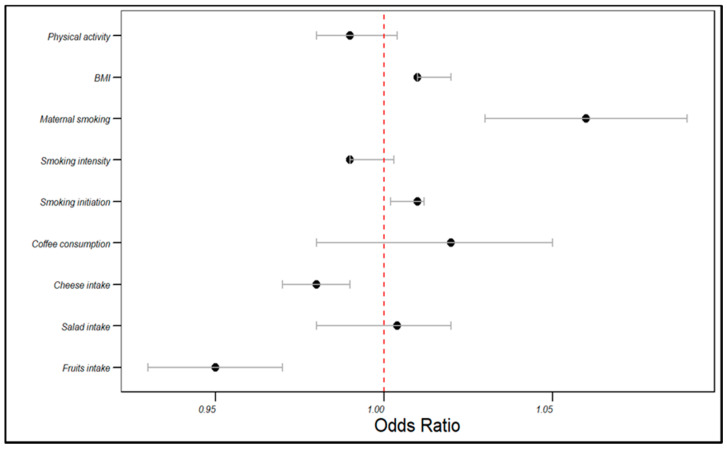
The influences of dietary patterns and lifestyle factors: on the angina risk in the UKB cohort.

In the UKB cohort, the genetically estimated fruit intake was associated with a decreased risk of angina (OR = 0.95, 95% CI: 0.93–0.97, *p* < 0.001), as was cheese intake (OR = 0.98, 95% CI: 0.97–0.99, *p* < 0.001). Conversely, smoking initiation (OR = 1.01, 95% CI: 1.002–1.012, *p* < 0.001) and maternal smoking (OR = 1.06, 95% CI: 1.03–1.09, *p* < 0.001) were associated with an increased angina risk. BMI was positively correlated with the angina risk (OR = 1.01, 95% CI: 1.01–1.02, *p* < 0.001). However, salad intake, coffee consumption, smoking intensity, and physical activity did not significantly influence the angina risk (*p* > 0.05). The rest of the associations are displayed in Table 2 and visualized in Figure 2.

#### 3.2.2. Angina Risk in the FinnGen Cohort

In the FinnGen cohort, the genetically estimated fruit intake was associated with a significantly reduced angina risk (OR = 0.30, 95% CI: 0.17–0.53, *p* < 0.001), as was the salad intake (OR = 0.31, 95% CI: 0.12–0.55, *p* = 0.003). Conversely, smoking initiation and intensity were associated with a higher angina risk (OR = 1.20, 95% CI: 1.04–1.37, *p* = 0.04; OR = 1.15, 95% CI: 1.04–1.26, *p* = 0.05, respectively), and BMI showed a positive relationship with the angina risk (OR = 1.31, 95% CI: 1.18–1.47, *p* < 0.001).

**Figure 3:**
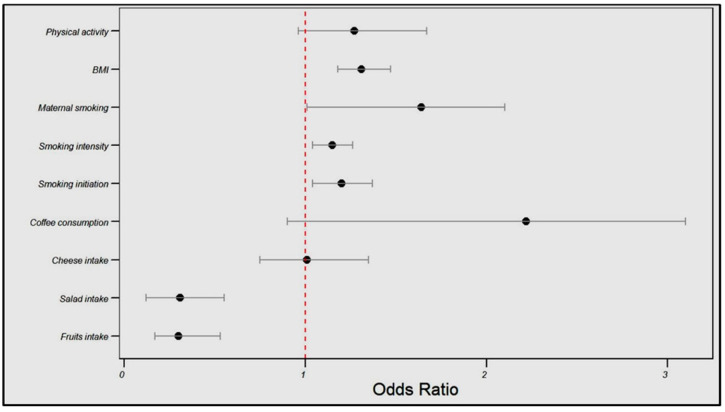
Influences of dietary patterns and lifestyle factors on angina risk in the FinnGen cohort.

No significant associations were found between cheese intake, coffee consumption, maternal smoking, or physical activity and angina risk (see Table 3 and Figure 3).

Both UKB and FinnGen cohorts showed consistent associations between fruit intake (protective), smoking initiation, and BMI (risk factors) with angina. However, salad intake was protective only in FinnGen, cheese intake was protective only in UKB, and smoking intensity was significant only in FinnGen.

## 4. Discussion

By harnessing the robust analytical framework of MR and data from two large population-based cohorts, the UK Biobank and FinnGen, the present investigation offers novel insights into the potential causal roles of various modifiable risk factors in the development of angina pectoris. Our findings shed light on the complex interplay between dietary patterns, smoking behaviors, adiposity, and physical activity and their associations with the risk of this prevalent cardiovascular condition.

Across both cohorts, increased fruit and salad intakes emerged as protective factors against angina risk, corroborating the cardioprotective effects of diets rich in fruits and vegetables observed in previous observational studies [25]. The beneficial impacts of these dietary components may be attributed to their high contents of antioxidants, such as polyphenols and vitamins, which can mitigate oxidative stress and inflammation, which are key drivers of atherosclerosis progression [26,27]. Furthermore, the fiber and phytochemicals found in fruits and vegetables may exert favorable effects on the lipid profile, blood pressure, and endothelial function, collectively contributing to a reduced risk of CAD and its clinical manifestations, including angina [28,29].

Conversely, our findings consistently implicate smoking initiation and intensity as causal risk factors for angina across both cohorts, aligning with the well-established detrimental effects of smoking on cardiovascular health [30]. Cigarette smoke exposure promotes oxidative stress, endothelial dysfunction, and inflammation, accelerating the progression of atherosclerotic lesions and increasing the risk of plaque rupture and thrombosis, which can culminate in angina or acute coronary events [31]. Additionally, smoking may exacerbate other risk factors, such as dyslipidemia and insulin resistance, further compounding the cardiovascular risk [32].

Our study also highlights the causal role of elevated BMI, a proxy for overall adiposity, in increasing the angina risk. This is consistent with the extensive body of literature linking obesity to CAD and its complications [33,34]. Excess adipose tissue, particularly visceral fat, contributes to a pro-inflammatory state and conditions associated with metabolic dysfunction, including dyslipidemia, insulin resistance, and hypertension, all of which are well-established risk factors for atherosclerotic cardiovascular disease [35]. Furthermore, obesity may exacerbate myocardial ischemia through various mechanisms such as increased myocardial oxygen demand, impaired coronary vasodilation, and endothelial dysfunction [36].

Discrepancies between UKB and FinnGen results may be due to differences in genetic backgrounds, dietary patterns, and study designs. These variations highlight the importance of cross-population studies in validating causal relationships between lifestyle factors and disease outcomes.

The causal pathways linking the studied factors to angina risk likely involve multiple mechanisms. Fruit and vegetable intake may reduce risk through antioxidant effects, improving endothelial function and reducing oxidative stress. Their fiber content may also lower cholesterol levels and improve glucose metabolism. Smoking may increase risk by promoting endothelial dysfunction, inflammation, and atherosclerosis. BMI’s impact could be mediated through increased systemic inflammation, insulin resistance, and altered lipid metabolism. While not showing a significant direct effect in this study, physical activity may indirectly influence angina risk by modulating these factors. This complex interplay underscores the multifaceted nature of angina pathophysiology and highlights potential preventive strategies.

It is important to interpret the odds ratios (ORs) and confidence intervals (CIs) in this study with caution, particularly for small effect sizes. For instance, the OR of 1.01 for smoking initiation in the UKB cohort, while statistically significant, represents only a marginal increase in risk. However, even small effect sizes can have substantial public health implications when considering population-level impacts. The clinical significance of these findings should be viewed in the context of lifelong exposures and potential cumulative effects. Moreover, the consistency of findings across different cohorts and their alignment with previous research strengthen the relevance of these results, despite some effect sizes being small.

Our findings both support and extend previous research. The protective effect of fruit intake aligns with observational studies like the EPIC-Heart study, which found inverse associations between fruit consumption and coronary heart disease risk. However, our study provides stronger causal evidence. The association between smoking and angina risk corroborates findings from the INTERHEART study, but our MR approach minimizes potential confounding. Regarding BMI, our results are consistent with a large-scale MR study that found causal effects of BMI on coronary artery disease [37]. Interestingly, our null finding for physical activity contrasts with observational studies suggesting protective effects, such as the Nurses’ Health Study. This discrepancy may be due to limitations in genetic instruments for physical activity or the complex interplay between physical activity, BMI, and cardiovascular health.

Notably, our findings reveal population-specific differences in the observed associations, highlighting the potential influences of genetic, environmental, and cultural factors on the interplay between risk factors and disease outcomes. For instance, while the genetically estimated high cheese intake appeared to be protective against angina in the UKB cohort, no significant association was detected in the FinnGen population. These disparities may reflect underlying differences in dietary patterns, gut microbiome compositions, or genetic predispositions that modulate the health effects of specific dietary components [38,39]. Additionally, the lack of association between physical activity and angina risk in both cohorts warrants further investigation, as previous studies have reported inconsistent findings, which is potentially attributable to variations in assessment methods, intensity levels, and the timing of exposure [40,41].

This study’s findings have potential policy implications. The protective effect of fruit intake supports dietary guidelines promoting fruit consumption and policies to improve fruit accessibility. The causal link between smoking and angina risk underscores the importance of robust smoking cessation programs. The BMI–angina association highlights the need for comprehensive obesity prevention strategies. While physical activity showed no direct causal effect, its potential indirect benefits suggest it should remain a key component of cardiovascular health promotion. These findings support an integrated approach to cardiovascular disease prevention, combining dietary interventions, smoking cessation, and weight management programs.

The generalizability of these findings to non-European populations warrants further investigation, given potential variations in genetic architecture, lifestyle factors, and environmental exposures across ethnic groups [42]. Future research should prioritize diverse cohorts, including populations of African, Asian, and Hispanic descent, to validate and extend these findings. Trans-ethnic meta-analyses could help identify shared and population-specific causal risk factors for angina [43]. Such investigations may uncover novel risk factors or differential effects across populations, contributing to more tailored prevention strategies globally.

While the strengths of the current study lie in its robust methodological approach and the integration of data from two large, well-characterized cohorts, it is essential to acknowledge the limitations in the generalizability of the findings. The UKB and FinnGen cohorts are predominantly composed of individuals of European ancestry, raising questions about the applicability of the results to populations with different genetic backgrounds, lifestyles, and environmental exposures. Future research should aim to incorporate more diverse populations to further validate and extend these findings. Additionally, the binary nature of the angina outcome may not capture the full spectrum of disease severity.

It is also important to recognize the potential limitations of this study, such as the reliance on summary-level data, which may preclude exploring the non-linear or time-varying effects of the exposures on the angina risk. Additionally, while MR is a powerful tool for causal inference, it relies on several key assumptions, including the validity of the genetic instruments and the absence of horizontal pleiotropy [4]. While I used methods robust to sample overlap, potential biases could still arise from overlap between exposure and outcome GWAS. Furthermore, the use of genetic variants as proxies for exposures may not fully capture their lifelong effects. Further research, incorporating complementary analytical approaches and individual-level data, is warranted to corroborate and extend our findings.

## 5. Conclusions

This study provides robust evidence for the causal roles of various modifiable risk factors in the development of angina pectoris. These findings underscore the potential benefits of dietary interventions promoting increased fruit and vegetable consumption, as well as the importance of smoking cessation and weight management in mitigating angina risk. However, the generalizability of these findings to populations with different genetic backgrounds, lifestyles, and environmental exposures remains to be established, given the predominance of individuals of European ancestry in the UKB and FinnGen cohorts. By elucidating the causal pathways underlying these associations, our results not only contribute to a deeper understanding of the etiology of angina but also could be used to inform the development of targeted preventive strategies and personalized treatment approaches, ultimately aiming to alleviate the substantial burden imposed by this condition on individuals and healthcare systems worldwide. These findings underscore the potential for targeted dietary and lifestyle interventions, such as promoting increased fruit and vegetable consumption, implementing robust smoking cessation programs, and developing comprehensive weight management strategies, as practical approaches to reduce angina risk at both individual and population levels. Further research is needed to validate these findings in diverse populations, as the causal relationships between modifiable risk factors and angina may vary across different genetic backgrounds and environmental contexts.

## Figures and Tables

**Table 1:** A brief summary of the genetic risk factors assessed in terms of the exposures involved, number of associated SNPs, sample sizes, and populations/consortiums.

Exposure	No. of SNPs	Sample Size	Population/Consortium
Fruits	41	409,125	Pirastu et al. [14]
Salad	22	462,933	UKB
Cheese	65	451,486	UKB
Coffee	3	64,949	UKB
Smoking initiation	93	632,802	GSCAN
Smoking intensity	23	249,752	GSCAN
Maternal smoking	16	397,732	MRC-IEU
BMI	79	339,152	GIANT
Number of days/weeks of vigorous physical activity	11	440,512	UKB

**Table 2:** Associations of risk factors with angina development in the UKB cohort. Bold indicates the significance level.

Risk Factor	Odds Ratio (OR) (95% CI)	*p* Value
Fruit intake	0.95 (0.93–0.97)	**<0.001**
Salad intake	1.004 (0.98–1.02)	0.694
Cheese intake	0.98 (0.97–0.99)	**<0.001**
Coffee consumption	1.02 (0.98–1.05)	0.288
Smoking initiation	1.01 (1.002–1.012)	**<0.001**
Smoking intensity	0.99 (0.99–1.003)	0.711
Maternal smoking	1.06 (1.03–1.09)	**<0.001**
BMI	1.01 (1.01–1.02)	**<0.001**
Physical activity	0.99 (0.98–1.004)	0.319

**Table 3:** Associations of risk factors with angina development in the FinnGen cohort. Bold indicates the significance level.

Risk Factor	OR (95% CI)	*p* Value
Fruit intake	0.30 (0.17–0.53)	**<0.001**
Salad intake	0.31 (0.12–0.55)	**0.003**
Cheese intake	1.01 (0.75–1.35)	0.950
Coffee consumption	2.22 (0.90–3.10)	0.243
Smoking initiation	1.20 (1.04–1.37)	**0.04**
Smoking intensity	1.15 (1.04–1.26)	**0.05**
Maternal smoking	1.64 (1.01–2.10)	0.350
BMI	1.31 (1.18–1.47)	**<0.001**
Physical activity	1.27 (0.96–1.67)	0.356

## Data Availability

The data utilized in this study are publicly available upon request to the respective consortia and cohort committees. The genetic data for fruit intake were obtained from Pirastu et al. (sample size 409,125) [14]. Data on salad intake (sample size 462,933), cheese intake (sample size 451,486), coffee consumption (sample size 64,949), and physical activity (sample size 440,512) were accessed from the UK Biobank [9,15,23]. Smoking initiation (sample size 632,802) and smoking intensity (sample size 249,752) data were obtained from the GWAS and Sequencing Consortium of Alcohol and Nicotine use (GSCAN) [16]. Maternal smoking data (sample size 397,732) were accessed from the MRC-IEU consortium [22]. BMI data (sample size 339,152) were obtained from the Genetic Investigation of Anthropometric Traits (GIANT) consortium [17]. Interested researchers can obtain these data by following the standard permissions protocols through the respective consortia and cohorts, including the UK Biobank Data Access Committee [9] and the FinnGen Data Access Committee [10]. The authors did not have any special access privileges beyond what would be available to other approved research groups. For this specific study, only summary-level statistics were accessed and analyzed by the authors. The Supplementary Materials can be downloaded directly at: https://www.mdpi.com/article/10.3390/life14070905/s1.

## Supplementary Materials

The following supporting information can be downloaded at: https://www.mdpi.com/article/10.3390/life14070905/s1, Table S1: Sensitivity analysis: heterogeneity assessment; Supplementary materials include Table S1, which presents a sensitivity analysis for heterogeneity assessment of various factors (e.g., fruits, salad, cheese, coffee consumption, smoking behaviors, BMI, and physical activity) in relation to angina. The table reports *p*-values for both MR Egger and inverse variance weighted methods. Additionally, detailed SNP information is provided for the exposure variables, including fruits consumption and BMI. These SNP tables contain data on chromosomal positions, effect sizes, standard errors, *p*-values, sample sizes, SNP IDs, and allele information for the genetic variants associated with each exposure.
