# Supplementary material for: Exploring the Genetic Roles of Diet and Other Modifiable Risk Factors in the Risk of Angina: A Causal Investigation Using Mendelian Randomization in UK Biobank and FinnGen Cohorts
Source: Life (Basel). 2024 Jul 20;14(7):905. doi: 10.3390/life14070905 (PMC11278461; doi:10.3390/life14070905)
Supplement: Supplementary file 1 [file life-14-00905-s001.zip › life-3100821-supplementary.pdf]

**Table S1.** Sensitivity analysis: heterogeneity assessment	
**Angina**	**P value**
Fruits	
MR Egger	0.0001228
Inverse variance weighted	0.000001613
Salad	
MR Egger	0.1741
Inverse variance weighted	0.01461
Cheese	
MR Egger	0.04
Inverse variance weighted	0.025
Coffee	
MR Egger	0.5842
Inverse variance weighted	0.4808
Smoking initiation	
MR Egger	0.0113
Inverse variance weighted	0.013
Smoking intensity	
MR Egger	0.1747
Inverse variance weighted	0.06562
Maternal smoking	
MR Egger	0.004914
Inverse variance weighted	0.005614
Body mass index (BMI)	
MR Egger	0.000287
Inverse variance weighted	0.00004804
Physical activity	
MR Egger	0.00009118
Inverse variance weighted	0.000001455

SNPs.exposure_fruits

chr.exposure	pos.exposure	beta.exposure	se.exposure	pval.exposure	samplesize.exposure	id.exposure	SNP	effect_allele.exposure	other_allele.exposure
1	72729142	-0.0108275	0.00139	6.72357E-15	409125	ebi-a-GCST90096909	rs1620977	G	A
1	98327133	-0.0109126	0.00175267	4.77661E-10	409125	ebi-a-GCST90096909	rs75641275	C	A
1	241055830	-0.00711357	0.00123694	8.87483E-09	409125	ebi-a-GCST90096909	rs261807	T	C
1	72128231	0.00728032	0.0012325	3.4849E-09	409125	ebi-a-GCST90096909	rs12120761	A	G
1	91190854	0.00825058	0.00150167	3.92329E-08	409125	ebi-a-GCST90096909	rs114328297	G	T
1	204593696	0.00940866	0.00149147	2.82091E-10	409125	ebi-a-GCST90096909	rs12046747	A	G
2	100985378	0.00819175	0.00122918	2.65766E-11	409125	ebi-a-GCST90096909	rs6542942	T	G
2	145734636	-0.00796269	0.00143262	2.72678E-08	409125	ebi-a-GCST90096909	rs2918593	C	T
2	170944488	-0.00792566	0.00128115	6.15673E-10	409125	ebi-a-GCST90096909	rs13018443	C	T
2	60225873	-0.00747933	0.00123386	1.34664E-09	409125	ebi-a-GCST90096909	rs6545772	T	C
2	60713235	-0.00782952	0.00124396	3.09329E-10	409125	ebi-a-GCST90096909	rs10189857	G	A
3	18763543	-0.00927969	0.00137239	1.36395E-11	409125	ebi-a-GCST90096909	rs4269101	G	T
3	43941406	0.00823553	0.00142633	7.74426E-09	409125	ebi-a-GCST90096909	rs11720884	G	A
3	147239337	0.00692187	0.00126638	4.60638E-08	409125	ebi-a-GCST90096909	rs57499472	C	T
4	2901600	0.0075463	0.00132878	1.35391E-08	409125	ebi-a-GCST90096909	rs7678161	T	C
4	37175523	0.00680471	0.00123338	3.44596E-08	409125	ebi-a-GCST90096909	rs1648404	T	C
6	25422369	0.0111688	0.00179717	5.14363E-10	409125	ebi-a-GCST90096909	rs6921589	C	A

6	98547979	0.00894444	0.00124014	5.49541E-13	409125	ebi-a-GCST90096909	rs9385269	T	C
7	132716502	-0.00779718	0.00131558	3.08894E-09	409125	ebi-a-GCST90096909	rs7808471	C	T
7	153485282	-0.00739951	0.00123574	2.12545E-09	409125	ebi-a-GCST90096909	rs2533273	A	C
7	24590331	0.00898635	0.00158475	1.42367E-08	409125	ebi-a-GCST90096909	rs2529055	G	A
7	2110346	0.0130461	0.00160593	4.52168E-16	409125	ebi-a-GCST90096909	rs3823624	C	T
8	144239099	0.00848131	0.0013236	1.47673E-10	409125	ebi-a-GCST90096909	rs11787024	C	T
10	126691471	-0.00996027	0.00164879	1.53204E-09	409125	ebi-a-GCST90096909	rs4348846	A	G
10	22098701	0.0129685	0.00136976	2.86022E-21	409125	ebi-a-GCST90096909	rs10828266	G	A
10	65191645	0.00702197	0.00123167	1.18982E-08	409125	ebi-a-GCST90096909	rs7924036	T	G
11	66292908	-0.01041	0.00134177	8.60003E-15	409125	ebi-a-GCST90096909	rs10896126	G	A
11	43619953	0.00868315	0.00124121	2.63937E-12	409125	ebi-a-GCST90096909	rs4755203	A	G
11	61514821	0.0111502	0.00178692	4.37855E-10	409125	ebi-a-GCST90096909	rs4963390	G	A
12	108618630	0.00964985	0.00140017	5.50427E-12	409125	ebi-a-GCST90096909	rs3764002	T	C
13	30883339	0.00682044	0.00124519	4.31549E-08	409125	ebi-a-GCST90096909	rs4769824	T	C
14	72170969	0.00692982	0.00123728	2.13304E-08	409125	ebi-a-GCST90096909	rs4140799	A	G
14	22038125	-0.0172135	0.00204232	3.50429E-17	409125	ebi-a-GCST90096909	rs34162196	T	C
14	77433198	0.0069661	0.00123928	1.89762E-08	409125	ebi-a-GCST90096909	rs10129747	G	A
16	52106298	0.00692086	0.00126444	4.41337E-08	409125	ebi-a-GCST90096909	rs1582323	G	A
16	73602926	-0.00676772	0.00123269	4.01467E-08	409125	ebi-a-GCST90096909	rs862227	G	A

**17**	56419228	0.00947428	0.00166181	1.18979E-08	409125	ebi-a-GCST90096909	rs62084586	C	T
**18**	57850583	0.00802209	0.00145617	3.60811E-08	409125	ebi-a-GCST90096909	rs17175518	A	C
**18**	60223017	0.00776009	0.0013195	4.07587E-09	409125	ebi-a-GCST90096909	rs12172847	A	G
**18**	21146085	0.008415	0.00123459	9.35837E-12	409125	ebi-a-GCST90096909	rs12457261	T	C
**19**	45411941	0.0158744	0.00170151	1.06267E-20	409125	ebi-a-GCST90096909	rs429358	C	T

SNPs.exposure.BMI

pval.exposure	samplesize.exposure	chr.exposure	se.exposure	beta.exposure	pos.exposure	id.exposure	SNP	effect_allele.exposure	other_allele.exposure
**2.18E-08**	339152	1	0.003	-0.0168	47684677	ieu-a-2	rs977747	G	T
**4.57E-11**	339065	1	0.0031	0.0201	78048331	ieu-a-2	rs17381664	C	T
**5.06E-14**	313621	1	0.0087	0.0659	110082886	ieu-a-2	rs7550711	T	C
**5.45E-10**	338768	1	0.0029	0.0181	201784287	ieu-a-2	rs2820292	C	A
**1.88E-28**	338123	1	0.003	0.0331	72837239	ieu-a-2	rs7531118	C	T
**2.29E-40**	339078	1	0.0037	0.0497	177889480	ieu-a-2	rs543874	G	A
**2.12E-13**	318585	1	0.0031	-0.0227	49589847	ieu-a-2	rs657452	G	A
**1.43E-13**	337797	1	0.003	0.0221	96924097	ieu-a-2	rs11165643	T	C
**4.98E-08**	339157	2	0.0038	0.0209	164567689	ieu-a-2	rs1460676	C	T
**4.77E-09**	338993	2	0.003	0.0175	181550962	ieu-a-2	rs1528435	T	C
**4.36E-12**	339033	2	0.0033	-0.0228	59305625	ieu-a-2	rs1016287	C	T
**1.24E-08**	218359	2	0.0049	0.0279	142959931	ieu-a-2	rs2890652	C	T
**5.44E-54**	333169	2	0.0039	0.0604	632348	ieu-a-2	rs13021737	G	A
**1.97E-08**	339118	2	0.0029	0.0164	227034499	ieu-a-2	rs6713510	A	G
**8.07E-26**	338829	2	0.0029	0.0309	25150296	ieu-a-2	rs10182181	G	A
**8.92E-09**	235995	2	0.0036	0.0207	58975143	ieu-a-2	rs12986742	C	T
**3.41E-08**	333383	2	0.0038	0.0211	208255518	ieu-a-2	rs17203016	G	A
**4.73E-11**	338186	2	0.0033	-0.0214	213413231	ieu-a-2	rs7599312	A	G
**1.93E-08**	294030	3	0.0033	0.0183	81792112	ieu-a-2	rs3849570	A	C
**8.02E-10**	338525	3	0.003	0.0183	25106437	ieu-a-2	rs6804842	G	A
**1.35E-10**	331668	3	0.003	-0.0195	61236462	ieu-a-2	rs2365389	T	C
**1.42E-14**	339205	3	0.0038	0.029	85807590	ieu-a-2	rs13078960	G	T
**1.85E-10**	236091	3	0.0075	0.0478	141275436	ieu-a-2	rs16851483	T	G
**1.39E-24**	337708	3	0.0044	0.0448	185824004	ieu-a-2	rs1516725	C	T

**5.03E-09**	235884	4	0.0052	0.0304	77129568	ieu-a-2	rs17001654	G	C
**8.01E-41**	338185	4	0.003	0.0398	45175691	ieu-a-2	rs13130484	T	C
**1.06E-12**	335619	4	0.0066	0.0472	103188709	ieu-a-2	rs13107325	T	C
**6.25E-09**	311073	4	0.0063	-0.0365	145659064	ieu-a-2	rs11727676	C	T
**1.96E-17**	339082	5	0.003	-0.0254	75015242	ieu-a-2	rs2112347	G	T
**8.85E-09**	339153	5	0.0029	-0.0168	153537893	ieu-a-2	rs7715256	T	G
**2.54E-10**	337750	6	0.0033	0.0209	34828553	ieu-a-2	rs6457796	C	T
**4.52E-31**	339197	6	0.0038	0.0444	50865820	ieu-a-2	rs943005	T	C
**1.45E-08**	338988	6	0.0032	0.0183	40348653	ieu-a-2	rs2033529	G	A
**4.28E-08**	339026	6	0.0043	0.0236	137675541	ieu-a-2	rs13201877	G	A
**1.09E-09**	338058	6	0.0047	-0.0285	163033350	ieu-a-2	rs13191362	G	A
**7.2E-09**	339068	6	0.0034	0.0196	120185665	ieu-a-2	rs9374842	T	C
**4.95E-08**	339072	6	0.0032	0.0175	108996963	ieu-a-2	rs3800229	T	G
**1.98E-10**	323308	7	0.0031	0.02	75163169	ieu-a-2	rs1167827	G	A
**9.46E-12**	339152	8	0.003	-0.0203	76650334	ieu-a-2	rs2060604	C	T
**2.22E-14**	339127	9	0.0032	0.0241	28412375	ieu-a-2	rs2183825	C	T
**6.36E-09**	338930	9	0.0029	-0.017	15634326	ieu-a-2	rs4740619	C	T
**4.32E-10**	337265	9	0.0029	-0.0182	120378483	ieu-a-2	rs1928295	C	T
**1.7E-08**	339115	9	0.003	-0.0169	111932342	ieu-a-2	rs6477694	T	C
**2.45E-10**	336886	9	0.003	-0.0188	129460914	ieu-a-2	rs10733682	G	A
**1.1E-12**	339198	10	0.0033	-0.0235	114758349	ieu-a-2	rs7903146	T	C
**1.27E-08**	338835	10	0.0067	0.0379	87410904	ieu-a-2	rs7899106	G	A
**2.19E-11**	338823	10	0.0037	0.0249	102395440	ieu-a-2	rs17094222	C	T
**3.47E-08**	333918	11	0.0033	-0.0185	43864278	ieu-a-2	rs2176598	C	T
**1.17E-17**	339006	11	0.003	0.0256	47650993	ieu-a-2	rs3817334	T	C
**5.44E-13**	337453	11	0.0029	0.0211	115022404	ieu-a-2	rs12286929	G	A

**6.67E-12**	339135	11	0.003	0.0206	8669437	ieu-a-2	rs10840100	G	A
**6.66E-30**	339035	11	0.0037	-0.0416	27684517	ieu-a-2	rs11030104	G	A
**5.11E-26**	339162	12	0.003	0.032	50247468	ieu-a-2	rs7138803	A	G
**1.22E-08**	328905	12	0.0053	-0.0304	122781897	ieu-a-2	rs11057405	A	G
**2.96E-08**	326858	13	0.0031	0.0172	79580919	ieu-a-2	rs1441264	A	G
**1.43E-10**	235969	13	0.0046	0.0295	28017270	ieu-a-2	rs9579083	C	G
**3.15E-13**	330001	13	0.0044	0.0324	54102206	ieu-a-2	rs12429545	A	G
**3.95E-09**	336023	13	0.0031	-0.0182	66205704	ieu-a-2	rs9540493	G	A
**6.05E-15**	338347	14	0.0035	0.0274	79940383	ieu-a-2	rs7144011	T	G
**1.4E-11**	338856	14	0.0033	-0.0221	25928179	ieu-a-2	rs10132280	A	C
**1.53E-18**	338200	15	0.0035	-0.0307	68104367	ieu-a-2	rs13329567	T	C
**4.52E-08**	338449	15	0.0029	-0.016	51748610	ieu-a-2	rs3736485	G	A
**3.9E-11**	330330	16	0.0037	0.0246	3599655	ieu-a-2	rs12448257	A	G
**3.94E-10**	235997	16	0.0039	0.0244	4015729	ieu-a-2	rs879620	T	C
**8.55E-11**	333191	16	0.0038	-0.0249	19941968	ieu-a-2	rs9926784	C	T
**6.58E-10**	338949	16	0.003	-0.0187	31011183	ieu-a-2	rs4889606	G	A
**2.17E-158**	333087	16	0.003	0.0803	53800954	ieu-a-2	rs1421085	C	T
**3.45E-25**	338077	16	0.003	0.0311	28889486	ieu-a-2	rs3888190	A	C
**3.64E-10**	339097	17	0.0029	-0.0183	78615571	ieu-a-2	rs12940622	A	G
**1.81E-08**	338903	17	0.0033	0.0184	5283252	ieu-a-2	rs1000940	G	A
**6.68E-59**	339006	18	0.0035	0.0562	57829135	ieu-a-2	rs6567160	C	T
**2E-13**	336765	18	0.005	-0.0371	58049656	ieu-a-2	rs17066856	C	T
**1.62E-08**	236115	18	0.0037	0.0209	21087531	ieu-a-2	rs891389	T	C
**1.92E-08**	338718	19	0.0033	0.0183	34304903	ieu-a-2	rs14810	G	C
**7.91E-19**	312079	19	0.0038	-0.0339	46180184	ieu-a-2	rs11672660	T	C
**1.59E-08**	234806	19	0.0043	0.0243	47602577	ieu-a-2	rs9304665	A	T

**7.79E-09**	334494	19	0.0034	-0.0196	18454825	ieu-a-2	rs17724992	G	A
**2.14E-08**	339045	20	0.0033	-0.0185	51087862	ieu-a-2	rs6091540	T	C
**1.6E-08**	337300	21	0.003	0.0169	40291740	ieu-a-2	rs2836754	C	T

SNPs.exposure.cheese

chr.exposure	se.exposure	pval.exposure	beta.exposure	pos.exposure	samplesize.exposure	id.exposure	SNP	effect_allele.exposure	other_allele.exposure
**1**	0.00327852	3.4E-08	0.0180965	4670487	451486	ukb-b-1489	rs78876700	A	G
**1**	0.00233731	1.8E-08	0.0131666	93676011	451486	ukb-b-1489	rs531358	T	C
**1**	0.00339704	4.2E-08	0.0186266	98546134	451486	ukb-b-1489	rs2802530	A	G
**1**	0.00241592	4.8E-08	-0.0131886	154295592	451486	ukb-b-1489	rs6685323	T	C
**2**	0.00244494	1.2E-09	0.0148578	24049453	451486	ukb-b-1489	rs2339928	A	G
**2**	0.00292518	4.4E-08	0.0160073	58433375	451486	ukb-b-1489	rs12475594	G	A
**2**	0.00234182	1E-31	0.0274418	45154689	451486	ukb-b-1489	rs504675	T	C
**2**	0.00340166	6.7E-11	0.0222011	136484232	451486	ukb-b-1489	rs72970243	A	G
**2**	0.00261651	3.9E-10	0.0163749	166299635	451486	ukb-b-1489	rs1514755	G	A
**3**	0.00328458	2.4E-09	0.0196018	161549397	451486	ukb-b-1489	rs79184944	A	T
**3**	0.00228781	1.2E-08	0.0130245	36915814	451486	ukb-b-1489	rs4296548	G	T
**3**	0.00316317	1.4E-08	-0.0179341	68410652	451486	ukb-b-1489	rs62245792	A	T
**3**	0.00827804	9.8E-09	-0.0474668	107637010	451486	ukb-b-1489	rs77742462	G	A
**3**	0.0022428	1E-10	-0.0144972	49890613	451486	ukb-b-1489	rs2352974	T	C
**3**	0.00567262	2.5E-08	0.0316294	194803510	451486	ukb-b-1489	rs6774906	C	A
**3**	0.00224121	2.9E-08	-0.0124361	56704919	451486	ukb-b-1489	rs4681981	A	C
**4**	0.00435152	2.2E-08	0.0243652	61251106	451486	ukb-b-1489	rs4860341	C	T
**4**	0.00306664	1.9E-11	-0.0205914	17788715	451486	ukb-b-1489	rs73096946	C	T
**4**	0.00425285	7E-12	-0.0291639	103188709	451486	ukb-b-1489	rs13107325	T	C
**4**	0.00225751	1.8E-08	-0.0127071	45182527	451486	ukb-b-1489	rs10938397	G	A
**4**	0.00258904	1.3E-08	0.0147298	170228542	451486	ukb-b-1489	rs4692708	C	A
**5**	0.00229427	2.4E-08	-0.0128007	87985295	451486	ukb-b-1489	rs26579	C	G
**5**	0.00227073	3.9E-08	-0.0124766	153597288	451486	ukb-b-1489	rs6873324	C	A
**6**	0.00250387	1.5E-08	0.0141706	4478474	451486	ukb-b-1489	rs9504123	C	A

**6**	0.00290699	2.5E-13	0.0212756	19028788	451486	ukb-b-1489	rs975303	G	A
**6**	0.00223591	1.6E-08	0.0126363	62630863	451486	ukb-b-1489	rs1931805	C	T
**7**	0.00249985	1.3E-09	0.0151797	140144414	451486	ukb-b-1489	rs113367286	T	C
**7**	0.00267855	4.5E-10	-0.0167006	2203808	451486	ukb-b-1489	rs34198643	T	C
**7**	0.00239456	9E-09	-0.0137634	115461436	451486	ukb-b-1489	rs12672200	A	G
**7**	0.00241153	1.4E-09	-0.0146166	132526871	451486	ukb-b-1489	rs9649582	T	A
**8**	0.00225388	2.1E-16	-0.0185161	9173358	451486	ukb-b-1489	rs7012814	A	G
**8**	0.00226925	3.6E-08	-0.0124974	144246027	451486	ukb-b-1489	rs7386207	T	C
**8**	0.00256186	2.7E-10	0.0161808	10187716	451486	ukb-b-1489	rs13257887	C	T
**9**	0.0034399	5.3E-10	0.0213573	88029000	451486	ukb-b-1489	rs3911016	G	T
**9**	0.002293	1.6E-08	0.0129596	124640841	451486	ukb-b-1489	rs4503172	T	C
**10**	0.004035	4.1E-08	-0.0221467	63682387	451486	ukb-b-1489	rs1806771	G	T
**10**	0.00497824	2.4E-08	0.0277726	106766879	451486	ukb-b-1489	rs73335955	C	T
**11**	0.00283438	7.2E-11	-0.0184695	65577516	451486	ukb-b-1489	rs10896050	T	G
**11**	0.00271432	1.1E-09	0.0165432	7946480	451486	ukb-b-1489	rs67238148	T	G
**11**	0.00227272	2.6E-12	0.0159029	43633645	451486	ukb-b-1489	rs7936836	A	C
**11**	0.00492718	4E-11	0.0325373	124942269	451486	ukb-b-1489	rs73024305	C	G
**11**	0.00281988	1.2E-08	-0.0160712	89943676	451486	ukb-b-1489	rs12786959	T	A
**12**	0.00255161	2.4E-08	-0.0142421	371786	451486	ukb-b-1489	rs524468	G	A
**12**	0.00226242	1.3E-08	-0.0128634	107291383	451486	ukb-b-1489	rs1024853	G	C
**12**	0.00230588	1.1E-08	-0.013175	22322789	451486	ukb-b-1489	rs7298331	C	A
**12**	0.00297985	2.8E-10	0.0187942	49927148	451486	ukb-b-1489	rs12296440	A	G
**12**	0.00257996	1.5E-08	0.0145932	121456616	451486	ukb-b-1489	rs61953351	T	G
**13**	0.00229156	6.7E-12	0.015728	58715219	451486	ukb-b-1489	rs1073242	A	G
**13**	0.0032085	3.6E-08	-0.0176745	89816082	451486	ukb-b-1489	rs11620149	C	T
**14**	0.00228956	1.8E-08	-0.0128806	30122409	451486	ukb-b-1489	rs17115145	T	C

**15**	0.00270974	1.5E-09	0.0163794	83481880	451486	ukb-b-1489	rs35270670	G	A
**15**	0.00232683	3.5E-11	0.0154088	68080886	451486	ukb-b-1489	rs4776970	T	A
**16**	0.00340698	6.8E-09	0.0197476	7744180	451486	ukb-b-1489	rs12447542	A	G
**16**	0.00262279	2.2E-10	0.016654	1252369	451486	ukb-b-1489	rs61734410	T	C
**16**	0.00229986	1.4E-09	-0.0139355	28535834	451486	ukb-b-1489	rs62034322	A	G
**16**	0.00252205	9.9E-09	0.0144554	71992817	451486	ukb-b-1489	rs71386942	A	C
**16**	0.00229169	1.5E-09	0.0138485	30918487	451486	ukb-b-1489	rs11649653	G	C
**17**	0.00324329	7.9E-10	0.0199382	46675977	451486	ukb-b-1489	rs919109	C	G
**17**	0.00256857	3.7E-11	0.0169937	61998469	451486	ukb-b-1489	rs2854175	A	C
**17**	0.00304376	3.6E-12	-0.0211557	44794558	451486	ukb-b-1489	rs12951057	G	C
**18**	0.00223594	2.6E-14	0.0170282	21143739	451486	ukb-b-1489	rs2960578	G	T
**18**	0.00225868	9.5E-09	0.0129646	44829435	451486	ukb-b-1489	rs1434511	T	C
**20**	0.00241026	4.4E-17	-0.0202495	35528475	451486	ukb-b-1489	rs1291145	C	T
**20**	0.00239492	3.3E-08	0.013226	51296552	451486	ukb-b-1489	rs6126641	A	G
**22**	0.00364674	1.1E-11	0.0247617	41992169	451486	ukb-b-1489	rs62236533	A	G

SNPs.exposure.coffee

pos.exposure	samplesize.exposure	se.exposure	chr.exposure	pval.exposure	beta.exposure	id.exposure	SNP	effect_allele.exposure	other_allele.exposure
**17287269**	64949	0.00258921	7	2.9E-13	0.0188985	ukb-b-9508	rs6968865	T	A
**7938226**	64949	0.00426414	9	1.4E-09	-0.0258238	ukb-b-9508	rs2065113	G	A
**75027880**	64949	0.00288014	15	7.3E-10	0.017741	ukb-b-9508	rs2472297	T	C

SNPs.exposure.maternal

pos.exposure	samplesize.exposure	pval.exposure	se.exposure	chr.exposure	beta.exposure	id.exposure	SNP	effect_allele.exposure	other_allele.exposure
**44097438**	397732	6.4E-14	0.0010749	1	-0.00806153	ukb-b-17685	rs12405972	T	G
**164928199**	397732	4.5E-08	0.00116502	2	0.00637328	ukb-b-17685	rs35566160	G	A
**140939110**	397732	1.1E-11	0.00105655	4	-0.00717316	ukb-b-17685	rs36072649	A	T
**50748173**	397732	3.4E-08	0.00105303	5	-0.00581087	ukb-b-17685	rs4865667	T	C
**26159356**	397732	1.5E-10	0.00122528	6	-0.00784343	ukb-b-17685	rs2183947	A	G
**32315613**	397732	4E-12	0.00106298	7	0.00737506	ukb-b-17685	rs10226228	G	A
**114951541**	397732	2E-08	0.0010297	7	-0.00577877	ukb-b-17685	rs62477310	C	T
**75196531**	397732	1.9E-08	0.00108172	7	-0.00607554	ukb-b-17685	rs794356	A	G
**93114414**	397732	1.4E-09	0.00124967	8	0.0075623	ukb-b-17685	rs7002049	C	T
**136468701**	397732	2.1E-13	0.00164225	9	0.0120528	ukb-b-17685	rs75596189	T	C
**14453010**	397732	3.9E-08	0.00124268	9	-0.00682946	ukb-b-17685	rs1323341	G	A
**104727304**	397732	2.3E-09	0.00147053	10	0.0087823	ukb-b-17685	rs7899608	T	C
**113678423**	397732	5.1E-09	0.0012016	11	0.00702278	ukb-b-17685	rs2428019	A	C
**78870803**	397732	2.3E-14	0.00122232	15	-0.00933094	ukb-b-17685	rs576982	T	C
**24798079**	397732	4.8E-09	0.00117285	16	-0.00686495	ukb-b-17685	rs12923476	A	G
**61984317**	397732	2.5E-14	0.00130401	20	-0.00993868	ukb-b-17685	rs6011779	T	C

SNPs.exposure.PA

samplesize.exposure	chr.exposure	beta.exposure	se.exposure	pos.exposure	pval.exposure	id.exposure	SNP	effect_allele.exposure	other_allele.exposure
**440512**	3	0.0281995	0.00435446	84966018	9.4E-11	ukb-b-151	rs382210	A	G
**440512**	4	0.0257299	0.00429482	113612872	2.1E-09	ukb-b-151	rs6533635	A	G
**440512**	6	-0.0279214	0.00428031	108927842	6.9E-11	ukb-b-151	rs2764261	G	A
**440512**	6	-0.0347546	0.00580657	26158079	2.2E-09	ukb-b-151	rs7749823	C	A
**440512**	7	0.0291439	0.00446067	35020280	6.4E-11	ukb-b-151	rs328900	T	C
**440512**	7	-0.0268435	0.00481883	8633758	2.5E-08	ukb-b-151	rs2189464	T	C
**440512**	7	-0.0321138	0.0042469	133581873	4E-14	ukb-b-151	rs6955240	A	G
**440512**	9	0.0250295	0.00426871	33791164	4.5E-09	ukb-b-151	rs2005617	C	T
**440512**	10	0.0273454	0.00463188	22032942	3.6E-09	ukb-b-151	rs7072776	G	A
**440512**	11	0.0253751	0.00436953	27792891	6.3E-09	ukb-b-151	rs1491872	T	C
**440512**	19	0.0388724	0.00572786	45411941	1.1E-11	ukb-b-151	rs429358	C	T

SNPs.exposure.salad

pos.exposure	samplesize.exposure	chr.exposure	se.exposure	beta.exposure	pval.exposure	id.exposure	SNP	effect_allele.exposure	other_allele.exposure
**153761750**	435435	1	0.00144212	-0.00800801	2.8E-08	ukb-b-1996	rs9427220	T	A
**231866480**	435435	1	0.00311517	0.017133	3.8E-08	ukb-b-1996	rs4083969	G	C
**25110415**	435435	3	0.00145086	0.0124765	8E-18	ukb-b-1996	rs7619139	A	T
**63738189**	435435	4	0.00143029	0.00797933	2.4E-08	ukb-b-1996	rs13102393	G	C
**137717681**	435435	5	0.00181338	0.0111744	7.2E-10	ukb-b-1996	rs17460017	T	A
**87822672**	435435	5	0.00143565	-0.00860795	2E-09	ukb-b-1996	rs2194027	A	T
**32379383**	435435	6	0.00212261	-0.0133033	3.7E-10	ukb-b-1996	rs3129962	A	G
**396321**	435435	6	0.00169411	-0.0102862	1.3E-09	ukb-b-1996	rs12203592	T	C
**30737591**	435435	6	0.00176637	-0.0126218	9E-13	ukb-b-1996	rs3095337	C	G
**92348945**	435435	6	0.00352774	-0.0197339	2.2E-08	ukb-b-1996	rs75248709	T	C
**35215670**	435435	7	0.00153327	0.00894101	5.5E-09	ukb-b-1996	rs57221424	G	C
**77730153**	435435	7	0.00185732	-0.0113438	1E-09	ukb-b-1996	rs62461186	C	A
**64618026**	435435	8	0.00156199	0.012474	1.4E-15	ukb-b-1996	rs790561	G	A
**83061099**	435435	8	0.00197644	-0.0108184	4.4E-08	ukb-b-1996	rs7821179	C	G
**128645617**	435435	9	0.00151342	-0.00916475	1.4E-09	ukb-b-1996	rs10819082	A	G
**22037809**	435435	10	0.00159025	0.0112719	1.4E-12	ukb-b-1996	rs6482190	G	A
**54933682**	435435	13	0.00180794	-0.0104258	8.1E-09	ukb-b-1996	rs1890012	G	T
**97011280**	435435	15	0.00166573	-0.00935	2E-08	ukb-b-1996	rs12908495	A	C
**31195279**	435435	16	0.00142564	0.00816444	1E-08	ukb-b-1996	rs1052352	T	C
**43854655**	435435	17	0.0014749	-0.00805142	4.8E-08	ukb-b-1996	rs34186148	C	G
**38106410**	435435	18	0.00142481	-0.00839985	3.7E-09	ukb-b-1996	rs4291983	A	C
**19049865**	435435	21	0.00157797	0.0087404	3E-08	ukb-b-1996	rs8130508	A	G

SNPs.exposure.smoking.initiation

chr.exposure	pos.exposure	beta.exposure	se.exposure	pval.exposure	samplesize.exposure	id.exposure	SNP	effect_allele.exposure	other_allele.exposure
1	44037685	0.0335118	0.0038983	8.12E-18	632802	ieu-b-4877	rs3001723	A	G
1	73766037	-0.0241444	0.00355604	1.14E-11	632802	ieu-b-4877	rs7555507	T	C
1	50625979	0.0260041	0.0043955	3.36E-09	632802	ieu-b-4877	rs6669839	T	C
1	91196176	-0.0222834	0.0035682	4.22E-10	632802	ieu-b-4877	rs12042107	C	T
1	66470206	0.0260573	0.00358602	3.61E-13	632802	ieu-b-4877	rs2186122	T	A
1	8481016	0.0214679	0.00361329	2.8E-09	632802	ieu-b-4877	rs301805	G	T
1	154205120	-0.0329992	0.00533915	6.52E-10	632802	ieu-b-4877	rs12025237	C	A
1	87905828	-0.0205467	0.00370828	3E-08	632802	ieu-b-4877	rs2050586	C	G
1	210304319	-0.0248139	0.00447842	3.03E-08	632802	ieu-b-4877	rs2046850	T	C
2	623976	0.0354486	0.00473279	6.73E-14	632802	ieu-b-4877	rs6728726	C	T
2	58171220	0.0205357	0.00365894	2.03E-08	632802	ieu-b-4877	rs78411160	C	A
2	182034448	0.0224483	0.00405809	3.16E-08	632802	ieu-b-4877	rs6433897	C	T
2	104088751	-0.0305098	0.00373855	3.36E-16	632802	ieu-b-4877	rs266047	A	G
2	226332033	-0.0252122	0.00443619	1.32E-08	632802	ieu-b-4877	rs4674993	G	A
2	45143175	0.0286801	0.00359627	1.5E-15	632802	ieu-b-4877	rs578584	T	A
2	137542847	0.0252442	0.00423093	2.43E-09	632802	ieu-b-4877	rs35702515	T	G
2	146143090	0.0360925	0.0035563	3.56E-24	632802	ieu-b-4877	rs13030994	A	G
2	162802993	0.0276329	0.00358234	1.25E-14	632802	ieu-b-4877	rs12474587	T	G
2	200937901	-0.027201	0.00492533	3.27E-08	632802	ieu-b-4877	rs2107300	G	C
2	60024857	0.0223996	0.0037281	1.88E-09	632802	ieu-b-4877	rs7585579	G	C
2	155682556	0.0239932	0.00356484	1.68E-11	632802	ieu-b-4877	rs1445649	C	T
3	85624131	-0.0313461	0.00368905	1.91E-17	632802	ieu-b-4877	rs6788098	T	A
3	50224225	-0.0233768	0.00375292	4.78E-10	632802	ieu-b-4877	rs12632110	G	A
3	75009019	-0.0270476	0.00457843	3.51E-09	632802	ieu-b-4877	rs11712680	C	A

**3**	117804154	0.0326217	0.00491232	3.12E-11	632802	ieu-b-4877	rs1154693	G	A
**3**	85985324	-0.0202716	0.00365268	2.83E-08	632802	ieu-b-4877	rs66680800	T	G
**3**	5724536	0.0197411	0.00356289	2.97E-08	632802	ieu-b-4877	rs1869243	C	T
**3**	85766025	0.0240472	0.00414237	6.32E-09	632802	ieu-b-4877	rs9835772	T	A
**4**	28473524	0.0237181	0.00403803	4.37E-09	632802	ieu-b-4877	rs962625	G	A
**4**	67825894	-0.025928	0.00429163	1.53E-09	632802	ieu-b-4877	rs993700	C	T
**4**	140927812	-0.0232512	0.00366263	2.14E-10	632802	ieu-b-4877	rs13145728	C	G
**4**	147797214	-0.0249918	0.00364155	6.65E-12	632802	ieu-b-4877	rs10001365	A	G
**4**	94052854	0.0207724	0.00358893	7.2E-09	632802	ieu-b-4877	rs1160685	G	C
**5**	60374912	-0.0240995	0.00407356	3.25E-09	632802	ieu-b-4877	rs6893752	G	A
**5**	103816655	-0.0332644	0.00502051	3.42E-11	632802	ieu-b-4877	rs12186738	T	G
**5**	154839646	0.0246617	0.00415673	3E-09	632802	ieu-b-4877	rs1385108	T	C
**5**	166989513	-0.0278417	0.00371058	6.08E-14	632802	ieu-b-4877	rs4044321	G	A
**5**	87756821	-0.0275284	0.00356881	1.22E-14	632802	ieu-b-4877	rs4352629	T	C
**5**	106834363	-0.0328856	0.00528628	5.02E-10	632802	ieu-b-4877	rs72789632	T	C
**6**	98748008	0.0277307	0.003986	3.47E-12	632802	ieu-b-4877	rs9401770	A	G
**6**	52916062	-0.0253208	0.00442796	1.08E-08	632802	ieu-b-4877	rs222449	T	A
**6**	108994161	0.0228121	0.00405809	1.93E-08	632802	ieu-b-4877	rs3800227	G	A
**6**	67405337	0.0206103	0.00355561	6.62E-09	632802	ieu-b-4877	rs10498846	T	C
**6**	111644332	-0.0410444	0.00483712	2.16E-17	632802	ieu-b-4877	rs240963	C	T
**7**	99185406	-0.0290467	0.00480127	1.44E-09	632802	ieu-b-4877	rs12333760	C	T
**7**	117523709	0.027069	0.00355741	2.75E-14	632802	ieu-b-4877	rs10233018	G	A
**7**	133589846	-0.0214194	0.00366263	5E-09	632802	ieu-b-4877	rs10279261	A	G
**7**	1889773	-0.0203218	0.00360938	1.75E-08	632802	ieu-b-4877	rs10260968	A	G
**7**	69735251	-0.024526	0.00404299	1.34E-09	632802	ieu-b-4877	rs12112638	G	A
**7**	1708080	-0.0247689	0.00355661	3.35E-12	632802	ieu-b-4877	rs4236259	G	T

**7**	3407568	-0.0232591	0.00373402	4.7E-10	632802	ieu-b-4877	rs2140114	T	C
**7**	96638267	-0.0220618	0.00373982	3.74E-09	632802	ieu-b-4877	rs3801289	C	A
**8**	27426077	-0.037618	0.0044613	3.42E-17	632802	ieu-b-4877	rs1565735	A	T
**8**	93201036	0.0264481	0.00388691	1.04E-11	632802	ieu-b-4877	rs1899896	T	C
**8**	59814666	-0.0268946	0.00355604	3.9E-14	632802	ieu-b-4877	rs13261666	T	G
**8**	65073605	0.0202808	0.00363668	2.43E-08	632802	ieu-b-4877	rs12545053	G	A
**8**	91995577	-0.0229645	0.00402823	1.18E-08	632802	ieu-b-4877	rs2631024	G	A
**9**	3014254	0.0219314	0.00356244	7.46E-10	632802	ieu-b-4877	rs4543592	C	T
**9**	86707289	0.0209482	0.00356645	4.16E-09	632802	ieu-b-4877	rs2378662	A	G
**9**	11070165	-0.0255148	0.00453171	1.81E-08	632802	ieu-b-4877	rs10114490	A	G
**10**	8803551	-0.0239554	0.00414505	7.35E-09	632802	ieu-b-4877	rs10905461	C	T
**10**	63674885	-0.0254601	0.00355815	8.26E-13	632802	ieu-b-4877	rs7921378	C	G
**10**	104563808	0.03937	0.0050491	6.27E-15	632802	ieu-b-4877	rs12356821	C	G
**10**	21766969	0.0262501	0.00372727	1.84E-12	632802	ieu-b-4877	rs10159545	G	C
**10**	125680419	-0.0205132	0.00370828	3.21E-08	632802	ieu-b-4877	rs9423279	G	C
**11**	112911004	0.0437914	0.00363668	2.71E-33	632802	ieu-b-4877	rs7938812	G	T
**11**	27679916	-0.0317863	0.00457843	3.77E-12	632802	ieu-b-4877	rs6265	T	C
**11**	85980958	0.0242377	0.00428466	1.56E-08	632802	ieu-b-4877	rs7929518	G	A
**11**	7950797	-0.0206091	0.00364321	1.55E-08	632802	ieu-b-4877	rs4523689	G	A
**12**	16748721	-0.0209298	0.00357892	4.85E-09	632802	ieu-b-4877	rs11057005	G	A
**12**	56508409	-0.0216913	0.00393413	3.58E-08	632802	ieu-b-4877	rs4759228	C	G
**12**	69655167	-0.0243756	0.00395946	7.31E-10	632802	ieu-b-4877	rs7969559	G	A
**12**	121389500	0.0285074	0.00492533	7.06E-09	632802	ieu-b-4877	rs1971318	T	C
**13**	100548329	-0.0255713	0.00433474	3.58E-09	632802	ieu-b-4877	rs7322872	T	C
**13**	38357471	-0.0211589	0.0035765	3.23E-09	632802	ieu-b-4877	rs3904512	A	G
**13**	66947124	-0.0195522	0.00355789	3.82E-08	632802	ieu-b-4877	rs9540729	T	A

**14**	29500130	-0.0249903	0.00454745	3.99E-08	632802	ieu-b-4877	rs76214862	C	A
**15**	83922387	-0.0292051	0.00452262	1.06E-10	632802	ieu-b-4877	rs12441907	A	C
**15**	47935843	0.0294151	0.00359095	2.64E-16	632802	ieu-b-4877	rs1435741	A	G
**16**	65604652	-0.0204704	0.00365894	2.26E-08	632802	ieu-b-4877	rs4785836	C	T
**16**	717085	-0.0247672	0.0041686	2.77E-09	632802	ieu-b-4877	rs7197072	T	C
**16**	87443734	-0.0216231	0.00358893	1.67E-09	632802	ieu-b-4877	rs1050847	T	C
**16**	17572674	-0.0238668	0.00436473	4.54E-08	632802	ieu-b-4877	rs4781977	C	T
**17**	7795972	-0.0201721	0.00360561	2.23E-08	632802	ieu-b-4877	rs11078713	G	A
**17**	30657058	-0.0207099	0.00365532	1.43E-08	632802	ieu-b-4877	rs7224742	T	C
**17**	2072949	0.0201357	0.00361066	2.43E-08	632802	ieu-b-4877	rs11658881	G	A
**18**	50026142	-0.0206935	0.00358602	7.97E-09	632802	ieu-b-4877	rs6508144	G	C
**18**	72535282	-0.0247725	0.00409477	1.43E-09	632802	ieu-b-4877	rs11872397	A	G
**18**	42632652	-0.0268885	0.00483712	2.75E-08	632802	ieu-b-4877	rs72896886	C	G
**19**	4474725	-0.0495575	0.00825958	1.94E-09	632802	ieu-b-4877	rs76608582	A	C
**20**	31175258	0.0225548	0.0038234	3.65E-09	632802	ieu-b-4877	rs1555445	T	A
**21**	40555561	0.0292897	0.00526909	2.76E-08	632802	ieu-b-4877	rs117143374	C	T
**22**	28781758	-0.019984	0.00366078	4.85E-08	632802	ieu-b-4877	rs134529	C	T

SNPs.exposure.smoking.intensity

chr.exposure	pos.exposure	beta.exposure	se.exposure	pval.exposure	samplesize.exposure	id.exposure	SNP	effect_allele.exposure	other_allele.exposure
1	154548521	-0.0652518	0.00924651	1.71E-12	225752	ieu-b-142	rs2072659	G	C
3	16872929	0.0336406	0.00590094	1.22E-08	260706	ieu-b-142	rs2084533	T	C
3	48935583	-0.0349579	0.0058102	1.82E-09	261833	ieu-b-142	rs7431710	A	G
4	67904931	0.0304766	0.00557353	4.5E-08	262450	ieu-b-142	rs787362	A	T
4	67053769	0.036064	0.00615782	4.67E-09	250822	ieu-b-142	rs11725618	C	T
6	26214473	-0.0308507	0.00553204	2.48E-08	257120	ieu-b-142	rs806798	C	T
7	32333642	-0.0492503	0.0057525	1.1E-17	262746	ieu-b-142	rs215600	A	G
8	27442127	0.0554895	0.00876271	2.44E-10	255628	ieu-b-142	rs73229090	A	C
8	64604218	-0.0408927	0.00619302	3.97E-11	261674	ieu-b-142	rs790564	C	A
8	42579203	0.0669394	0.00650172	9E-25	258370	ieu-b-142	rs58379124	C	T
9	136502369	-0.0578394	0.00704521	2.22E-16	256134	ieu-b-142	rs3025383	C	T
11	16377044	0.0389511	0.00596781	6.63E-11	258295	ieu-b-142	rs7951365	C	T
11	46465361	0.0598763	0.0105682	1.45E-08	258399	ieu-b-142	rs75494138	T	C
11	113448762	-0.0329314	0.00555848	3.14E-09	258763	ieu-b-142	rs7928017	A	C
15	59155050	-0.0367137	0.00641049	1.03E-08	214251	ieu-b-142	rs632811	G	A
15	78806023	0.182567	0.00588925	1E-200	255729	ieu-b-142	rs8034191	C	T
16	89772619	-0.0336223	0.00553496	1.27E-09	254249	ieu-b-142	rs4785587	A	G
16	52074530	-0.0318201	0.00556452	1.07E-08	259482	ieu-b-142	rs1579233	G	A
19	4060707	-0.0390041	0.00701668	2.68E-08	250059	ieu-b-142	rs895330	G	C
19	41353107	0.107205	0.00560367	1.1E-81	243952	ieu-b-142	rs56113850	C	T
19	41305530	-0.146992	0.0166972	1.33E-18	251880	ieu-b-142	rs34406232	A	C
20	61986949	0.0680938	0.00779553	2.47E-18	252983	ieu-b-142	rs2273500	C	T
20	31047533	0.0334848	0.00563586	2.76E-09	257430	ieu-b-142	rs2424888	A	G

SNPs.outcome.BMI

SNP	chr	pos	beta.outcome	se.outcome	samplesize.outcome
rs2820292	1	201784287	0.000707713	0.000428796	336683
rs7599312	2	213413231	-0.000276648	0.000483276	336683
rs2112347	5	75015242	-0.000288182	0.000443652	336683
rs17203016	2	208255518	-0.000572736	0.000530922	336683
rs12986742	2	58975143	0.000950038	0.000427551	336683
rs2890652	2	142959931	0.000349401	0.000576267	336683
rs17001654	4	77129568	0.00103208	0.000606127	336683
rs6457796	6	34828553	0.00103371	0.000479097	336683
rs891389	18	21087531	0.00113537	0.000446698	336683
rs3736485	15	51748610	0.000327634	0.000427899	336683
rs13021737	2	632348	0.000403626	0.000562964	336683
rs1516725	3	185824004	9.17E-05	0.00062084	336683
rs2060604	8	76650334	-0.000358926	0.000431926	336683
rs12429545	13	54102206	0.000346578	0.000638193	336683
rs17724992	19	18454825	-0.000725693	0.000481071	336683
rs977747	1	47684677	0.00011823	0.00043166	336683
rs2365389	3	61236462	-0.000394127	0.000432971	336683
rs6804842	3	25106437	0.00125914	0.000430281	336683
rs16851483	3	141275436	0.0005014	0.000856651	336683
rs10132280	14	25928179	-0.000462855	0.000465564	336683
rs12448257	16	3599655	-0.000379301	0.000519766	336683
rs2183825	9	28412375	0.000635145	0.000452757	336683
rs4889606	16	31011183	-0.000640339	0.000439744	336683
rs2176598	11	43864278	-0.00121477	0.000493877	336683
rs879620	16	4015729	7.13E-05	0.000438228	336683
rs14810	19	34304903	-9.23E-05	0.000454418	336683
rs17066856	18	58049656	-0.000374064	0.000738169	336683
rs543874	1	177889480	0.000683716	0.00052319	336683
rs11165643	1	96924097	2.16E-05	0.000432247	336683
rs7531118	1	72837239	0.000306438	0.000428534	336683
rs13107325	4	103188709	-0.000394269	0.000808593	336683
rs11030104	11	27684517	-0.00057295	0.000527428	336683
rs3800229	6	108996963	0.000674131	0.000472187	336683
rs13191362	6	163033350	-0.000526894	0.000643438	336683
rs943005	6	50865820	0.000850323	0.000568111	336683
rs2033529	6	40348653	0.000629966	0.000470472	336683
rs13078960	3	85807590	0.000636165	0.000530637	336683
rs13130484	4	45175691	-0.000202017	0.000429828	336683
rs6713510	2	227034499	-0.00116875	0.00042682	336683

**rs6477694**	9	111932342	-0.000322155	0.000445298	336683
**rs3888190**	16	28889486	-0.000501306	0.000432943	336683
**rs1000940**	17	5283252	-0.000817685	0.000464069	336683
**rs1421085**	16	53800954	-0.000172408	0.000433208	336683
**rs9304665**	19	47602577	0.00107696	0.000502178	336683
**rs9926784**	16	19941968	0.00110251	0.000549237	336683
**rs2836754**	21	40291740	-0.000509362	0.000442449	336683
**rs12940622**	17	78615571	-0.000628574	0.000427319	336683
**rs1441264**	13	79580919	0.000544564	0.00044143	336683
**rs1016287**	2	59305625	-0.000504133	0.00046466	336683
**rs1460676**	2	164567689	-2.2E-05	0.000586747	336683
**rs11727676**	4	145659064	-0.000678512	0.000721014	336683
**rs10840100**	11	8669437	0.000505762	0.000447381	336683
**rs3817334**	11	47650993	0.00108809	0.000431577	336683
**rs7899106**	10	87410904	-0.000670384	0.000971704	336683
**rs7903146**	10	114758349	0.00153693	0.000467334	336683
**rs7144011**	14	79940383	-0.0004916	0.000511821	336683
**rs4740619**	9	15634326	-0.00103625	0.000427095	336683
**rs13329567**	15	68104367	-0.000346656	0.000509875	336683
**rs10733682**	9	129460914	-0.000313267	0.000431647	336683
**rs9540493**	13	66205704	-0.000654641	0.000428566	336683
**rs11672660**	19	46180184	-0.000353972	0.000536311	336683
**rs7138803**	12	50247468	0.000378038	0.000440281	336683
**rs9579083**	13	28017270	0.000211429	0.000551614	336683
**rs3849570**	3	81792112	-0.000348028	0.000445576	336683
**rs1528435**	2	181550962	0.000898628	0.000438001	336683
**rs7550711**	1	110082886	-0.00123225	0.00134142	336683
**rs17381664**	1	78048331	0.000814648	0.000431802	336683
**rs7715256**	5	153537893	-0.000614363	0.000428992	336683
**rs13201877**	6	137675541	-0.0013359	0.000629658	336683
**rs1167827**	7	75163169	-5.62E-05	0.000428899	336683
**rs9374842**	6	120185665	0.00102095	0.000505562	336683
**rs12286929**	11	115022404	0.0013365	0.000425856	336683
**rs17094222**	10	102395440	0.000214518	0.00052041	336683
**rs10182181**	2	25150296	0.00103994	0.000424928	336683
**rs657452**	1	49589847	-0.000325082	0.000436971	336683
**rs11057405**	12	122781897	0.000374417	0.000690014	336683
**rs6567160**	18	57829135	0.000626106	0.000501426	336683
**rs6091540**	20	51087862	-0.000599319	0.000464789	336683
**rs1928295**	9	120378483	0.000341609	0.000428902	336683

SNPs.outcome.cheese

SNP	chr	pos	beta.outcome	se.outcome	samplesize.outcome
rs78876700	1	4670487	-0.000598031	0.000621522	336683
rs2960578	18	21143739	-0.000988512	0.000424882	336683
rs12447542	16	7744180	-0.000435247	0.000647596	336683
rs524468	12	371786	-0.000715377	0.000485397	336683
rs12786959	11	89943676	-1.57E-05	0.000534576	336683
rs10896050	11	65577516	-8.4E-05	0.000538246	336683
rs1024853	12	107291383	-0.000103251	0.000429956	336683
rs113367286	7	140144414	-0.00120423	0.000474363	336683
rs12296440	12	49927148	-0.000946635	0.00056942	336683
rs3911016	9	88029000	1.07E-05	0.000654008	336683
rs4503172	9	124640841	-0.000405099	0.000435326	336683
rs10938397	4	45182527	-0.000221884	0.000429566	336683
rs2352974	3	49890613	0.000335372	0.000426302	336683
rs4296548	3	36915814	9.98E-05	0.000435194	336683
rs6774906	3	194803510	-0.000988563	0.00108052	336683
rs4776970	15	68080886	7.5E-05	0.000442994	336683
rs34198643	7	2203808	9.34E-05	0.000509346	336683
rs7012814	8	9173358	0.00050484	0.000428435	336683
rs26579	5	87985295	-0.00111811	0.000436632	336683
rs4692708	4	170228542	7.35E-06	0.000491952	336683
rs62034322	16	28535834	-0.000271016	0.000436711	336683
rs71386942	16	71992817	0.000240933	0.000479786	336683
rs12951057	17	44794558	0.000596707	0.000575816	336683
rs1806771	10	63682387	-0.000328882	0.000765055	336683
rs2802530	1	98546134	-0.000659874	0.00064268	336683
rs12475594	2	58433375	-0.000323081	0.000557552	336683
rs504675	2	45154689	6.53E-05	0.000446422	336683
rs13107325	4	103188709	-0.000394269	0.000808593	336683
rs7298331	12	22322789	0.000392498	0.000438199	336683
rs62245792	3	68410652	-0.00101424	0.000599138	336683
rs79184944	3	161549397	-0.000575232	0.00062284	336683
rs7386207	8	144246027	0.000649516	0.000431059	336683
rs73335955	10	106766879	-0.00108943	0.000948825	336683
rs1291145	20	35528475	-0.000415678	0.000458314	336683
rs1434511	18	44829435	0.000512195	0.000429273	336683
rs35270670	15	83481880	-0.00064084	0.000515797	336683
rs17115145	14	30122409	0.000511054	0.000434749	336683
rs2854175	17	61998469	-0.00063823	0.000487572	336683
rs62236533	22	41992169	-0.000239749	0.000691955	336683

rs4860341	4	61251106	-0.00127092	0.000826725	336683
rs6685323	1	154295592	0.00102977	0.000459991	336683
rs1514755	2	166299635	3.37E-05	0.00049625	336683
rs531358	1	93676011	6.63E-05	0.000444164	336683
rs73096946	4	17788715	0.000842119	0.000587245	336683
rs7936836	11	43633645	-0.000248741	0.000431756	336683
rs67238148	11	7946480	-0.000178499	0.000513753	336683
rs6873324	5	153597288	0.000102432	0.000431781	336683
rs61953351	12	121456616	-0.000527202	0.000490241	336683
rs73024305	11	124942269	-0.0016485	0.000936273	336683
rs11620149	13	89816082	0.00037369	0.000610036	336683
rs919109	17	46675977	0.001198	0.000615595	336683
rs975303	6	19028788	-0.00137594	0.000550367	336683
rs11649653	16	30918487	-0.000486933	0.000436202	336683
rs77742462	3	107637010	0.00251917	0.00156063	336683
rs72970243	2	136484232	-0.00197021	0.000677082	336683
rs4681981	3	56704919	-0.000391798	0.00042652	336683
rs1931805	6	62630863	0.000528648	0.000425393	336683
rs61734410	16	1252369	-0.00105805	0.000500167	336683
rs12672200	7	115461436	-0.000942236	0.000455711	336683
rs1073242	13	58715219	-0.000255657	0.000435477	336683
rs9649582	7	132526871	0.000558421	0.000457695	336683
rs2339928	2	24049453	-0.00063084	0.00046447	336683
rs9504123	6	4478474	0.000142203	0.0004777	336683
rs6126641	20	51296552	-1.82E-05	0.000454509	336683

SNPs.outcome.coffee

SNP	chr	pos	beta.outcome	se.outcome	samplesize.outcome
**rs2065113**	9	7938226	0.000301638	0.000721106	336683
**rs2472297**	15	75027880	0.000360675	0.000479649	336683
**rs6968865**	7	17287998	0.00060091	0.000441953	336683

SNPs.outcome.fruits

SNP	chr	pos	beta.outcome	se.outcome	samplesize.outcome
rs1648404	4	37175523	0.000144952	0.000425585	336683
rs862227	16	73602926	0.000361177	0.000425307	336683
rs114328297	1	91190854	-0.000584604	0.000517607	336683
rs4269101	3	18763543	0.0012496	0.000473255	336683
rs429358	19	45411941	0.00316456	0.000584822	336683
rs13018443	2	170944488	0.000700592	0.000442612	336683
rs6542942	2	100985378	-0.000893272	0.000424196	336683
rs261807	1	241055830	0.000797222	0.000427191	336683
rs4140799	14	72170969	-0.000680643	0.000426606	336683
rs7808471	7	132716502	0.000359062	0.000453785	336683
rs3823624	7	2110346	-0.000885421	0.000554989	336683
rs2529055	7	24590331	-0.000681665	0.000547619	336683
rs11787024	8	144239099	-0.000432698	0.000455756	336683
rs4348846	10	126691471	0.00091706	0.000568234	336683
rs7924036	10	65191645	-9.75E-05	0.000425061	336683
rs10828266	10	22098701	-0.000430101	0.000472468	336683
rs10896126	11	66292908	-7.22E-05	0.000462339	336683
rs12457261	18	21146085	-0.000967134	0.000425204	336683
rs75641275	1	98327133	0.00131855	0.000605276	336683
rs12120761	1	72128231	-0.000360512	0.000425552	336683
rs10189857	2	60713235	0.00141705	0.000429421	336683
rs11720884	3	43941406	-0.00129849	0.000492145	336683
rs2918593	2	145734636	0.000469777	0.000494565	336683
rs12172847	18	60223017	-6.64E-05	0.000455395	336683
rs10129747	14	77433198	-0.00154013	0.000427252	336683
rs1620977	1	72729142	4.63E-05	0.000480753	336683
rs6545772	2	60225873	0.000188929	0.000425721	336683
rs57499472	3	147239337	-0.000925196	0.000436466	336683
rs4755203	11	43619953	-0.000477019	0.000428066	336683
rs4963390	11	61514821	6.74E-05	0.000618561	336683
rs9385269	6	98547979	-0.000570095	0.000427758	336683
rs62084586	17	56419228	0.000118469	0.000571936	336683
rs7678161	4	2901600	-0.00142087	0.000457905	336683
rs34162196	14	22038125	0.001297	0.000704617	336683
rs2533273	7	153485282	0.000291297	0.000426193	336683
rs6921589	6	25422369	-0.000117931	0.000617413	336683
rs4769824	13	30883339	-0.000446371	0.000429582	336683
rs12046747	1	204593696	-0.000514449	0.000514981	336683
rs3764002	12	108618630	0.000155734	0.000483409	336683

rs17175518	18	57850583	0.000615594	0.00050088	336683
rs1582323	16	52106298	0.000252986	0.00043605	336683

SNPs.outcome.initiation

SNP	chr	pos	beta.outcome	se.outcome	samplesize.outcome
rs2186122	1	66470206	0.000975875	0.000431105	336683
rs3001723	1	44037685	-0.000293327	0.000462999	336683
rs301805	1	8481016	0.000365964	0.000431528	336683
rs4352629	5	87756821	0.00158464	0.000427026	336683
rs13145728	4	140927812	-0.000889641	0.000437555	336683
rs993700	4	67825894	-0.000764013	0.000510396	336683
rs9401770	6	98748008	-0.000119653	0.00047959	336683
rs72896886	18	42632652	-2.43E-05	0.000570614	336683
rs134529	22	28781758	-0.000880927	0.000437542	336683
rs66680800	3	85985324	0.000143677	0.000433543	336683
rs240963	6	111644332	6.96E-05	0.00058068	336683
rs2631024	8	91995577	0.00023743	0.000486346	336683
rs4543592	9	3014254	-0.000234191	0.000425723	336683
rs13261666	8	59814666	0.000257185	0.000424813	336683
rs4044321	5	166989513	-0.00154385	0.00044305	336683
rs2140114	7	3407568	-0.000606451	0.00042606	336683
rs10233018	7	117523709	-0.000424613	0.000425309	336683
rs9540729	13	66947124	-0.000939147	0.000425135	336683
rs3904512	13	38357471	-0.000716125	0.000427557	336683
rs12474587	2	162802993	-1.38E-05	0.000427805	336683
rs7555507	1	73766037	-0.000720555	0.000425347	336683
rs2050586	1	87905828	-2.09E-05	0.000444205	336683
rs76214862	14	29500130	0.00115367	0.000545921	336683
rs7197072	16	717085	0.000299837	0.000508332	336683
rs7322872	13	100548329	0.000723738	0.000523741	336683
rs12441907	15	83922387	0.000902197	0.000541637	336683
rs7929518	11	85980958	0.00064111	0.000514693	336683
rs9423279	10	125680419	-0.000354304	0.000456037	336683
rs11057005	12	16748721	9.07E-05	0.000430968	336683
rs4785836	16	65604652	5.81E-05	0.000439679	336683
rs1050847	16	87443734	-0.000265371	0.000431615	336683
rs7224742	17	30657058	-0.00105715	0.000438231	336683
rs11658881	17	2072949	0.000127316	0.000431146	336683
rs11078713	17	7795972	-0.000310844	0.000431311	336683
rs7969559	12	69655167	-0.000462791	0.000473795	336683
rs7921378	10	63674885	0.000225566	0.000426365	336683
rs1555445	20	31175258	-4.43E-05	0.00046184	336683

**rs76608582**	19	4474725	-0.00188464	0.00104085	336683
**rs6728726**	2	623976	0.000247428	0.000561642	336683
**rs578584**	2	45143175	2.37E-05	0.000428425	336683
**rs7585579**	2	60024857	0.000185418	0.000429388	336683
**rs1160685**	4	94052854	-0.000302494	0.000427647	336683
**rs10001365**	4	147797214	-0.000388082	0.000436375	336683
**rs2378662**	9	86707289	-0.00041428	0.000427757	336683
**rs10279261**	7	133589846	0.000466055	0.000439005	336683
**rs12112638**	7	69735251	-0.000375103	0.000483095	336683
**rs10260968**	7	1889773	-0.000547284	0.000431178	336683
**rs1445649**	2	155682556	-5.42E-05	0.000426159	336683
**rs35702515**	2	137542847	-0.000293751	0.000505529	336683
**rs962625**	4	28473524	-0.000865464	0.000480592	336683
**rs72789632**	5	106834363	0.000239823	0.000634589	336683
**rs6893752**	5	60374912	3.19E-05	0.000484993	336683
**rs10905461**	10	8803551	-0.000668989	0.000487843	336683
**rs12545053**	8	65073605	0.000479781	0.000434317	336683
**rs1899896**	8	93201036	-0.000296495	0.000465744	336683
**rs12356821**	10	104563808	0.000601778	0.000605574	336683
**rs10159545**	10	21766969	0.000816657	0.000449048	336683
**rs4781977**	16	17572674	-6.73E-05	0.000513133	336683
**rs1154693**	3	117804154	0.000300848	0.000599508	336683
**rs2046850**	1	210304319	-0.00113929	0.000539384	336683
**rs12025237**	1	154205120	0.00019277	0.000640763	336683
**rs13030994**	2	146143090	0.000274269	0.000425061	336683
**rs11712680**	3	75009019	-0.000995153	0.000546074	336683
**rs4674993**	2	226332033	0.000300451	0.000532917	336683
**rs2107300**	2	200937901	-0.000324	0.000585838	336683
**rs4523689**	11	7950797	1.57E-05	0.000434503	336683
**rs6265**	11	27679916	-0.000831057	0.000541506	336683
**rs3801289**	7	96638267	-0.000506709	0.000450278	336683
**rs10498846**	6	67405337	3.41E-05	0.000426183	336683
**rs4236259**	7	1708080	0.00030856	0.000428985	336683
**rs3800227**	6	108994161	0.000539935	0.000488219	336683
**rs1971318**	12	121389500	-0.000610016	0.000583194	336683
**rs1565735**	8	27426077	-0.000724286	0.000533248	336683
**rs12632110**	3	50224225	6.34E-05	0.000448499	336683
**rs6508144**	18	50026142	-0.000177321	0.000430245	336683
**rs11872397**	18	72535282	-2.08E-05	0.000489617	336683
**rs9835772**	3	85766025	0.000363484	0.000495054	336683
**rs6669839**	1	50625979	7.37E-06	0.000523004	336683

**rs6433897**	2	182034448	0.000798217	0.000483013	336683
**rs1869243**	3	5724536	0.000518578	0.000426925	336683
**rs12042107**	1	91196176	-0.00045708	0.000427688	336683
**rs6788098**	3	85624131	-0.000106968	0.000439463	336683
**rs266047**	2	104088751	0.000211492	0.000425157	336683
**rs1385108**	5	154839646	0.000859865	0.000497482	336683
**rs222449**	6	52916062	-0.00055726	0.000534539	336683
**rs12333760**	7	99185406	-0.000939063	0.000573419	336683
**rs10114490**	9	11070165	-0.000297908	0.000546406	336683
**rs1435741**	15	47935843	-3.06E-05	0.000429128	336683
**rs7938812**	11	112911004	0.000253081	0.0004365	336683
**rs12186738**	5	103816655	-0.0016036	0.000606843	336683
**rs4759228**	12	56508409	0.000448502	0.000466074	336683
**rs117143374**	21	40555561	-0.000413374	0.000610375	336683

SNPs.outcome.intensity

SNP	chr	pos	beta.outcome	se.outcome	samplesize.outcome
rs806798	6	26214473	-0.000545835	0.000425675	336683
rs8034191	15	78806023	-0.00130866	0.000450095	336683
rs7431710	3	48935583	0.000270338	0.000446002	336683
rs895330	19	4060707	-0.000787167	0.000540014	336683
rs215600	7	32333642	-0.00114488	0.000444059	336683
rs4785587	16	89772619	-0.000112746	0.000429999	336683
rs2273500	20	61986949	0.000803306	0.000604737	336683
rs7951365	11	16377044	-0.000164032	0.000461533	336683
rs73229090	8	27442127	-0.000703116	0.000666	336683
rs58379124	8	42579203	-0.000122614	0.000506768	336683
rs790564	8	64604218	0.00054378	0.000476027	336683
rs56113850	19	41353107	0.000352045	0.000429712	336683
rs7928017	11	113448762	-0.000281969	0.000428473	336683
rs2084533	3	16872929	0.000103077	0.000461473	336683
rs75494138	11	46465361	6.93E-05	0.000817339	336683
rs632811	15	59155050	0.000161926	0.000452934	336683
rs1579233	16	52074530	0.000341727	0.000427876	336683
rs2424888	20	31047533	0.000442207	0.00044492	336683
rs2072659	1	154548521	0.000275808	0.000728556	336683
rs3025383	9	136502369	-0.00097703	0.000551917	336683
rs787362	4	67904931	0.000362605	0.000432253	336683
rs11725618	4	67053769	0.0004297	0.000492481	336683
rs34406232	19	41305530	-0.00132969	0.00131968	336683

SNPs.outcome.maternal

SNP	chr	pos	beta.outcome	se.outcome	samplesize.outcome
**rs36072649**	4	140939110	-0.000854807	0.000437672	336683
**rs2183947**	6	26159356	-0.00157884	0.000508377	336683
**rs6011779**	20	61984317	-0.00104769	0.00054188	336683
**rs10226228**	7	32315613	0.00121357	0.000440737	336683
**rs4865667**	5	50748173	-0.000349175	0.000436258	336683
**rs2428019**	11	113678423	0.000342283	0.000497032	336683
**rs1323341**	9	14453010	7.68E-06	0.000514846	336683
**rs12405972**	1	44097438	0.000251524	0.000446609	336683
**rs7899608**	10	104727304	0.000865964	0.000610209	336683
**rs794356**	7	75196531	-3.76E-05	0.000448348	336683
**rs7002049**	8	93114414	0.000961716	0.000516342	336683
**rs35566160**	2	164928199	-0.00106584	0.000483059	336683
**rs576982**	15	78870803	0.000824063	0.000506542	336683
**rs62477310**	7	114951541	-0.000351564	0.000426453	336683
**rs75596189**	9	136468701	0.00149838	0.000676426	336683
**rs12923476**	16	24798079	-0.000315886	0.000487148	336683

SNPs.outcome.PA

SNP	chr	pos	beta.outcome	se.outcome	samplesize.outcome
**rs7749823**	6	26158079	-0.000272833	0.000593111	336683
**rs7072776**	10	22032942	-0.000607205	0.000476485	336683
**rs6955240**	7	133581873	0.00108375	0.000435992	336683
**rs429358**	19	45411941	0.00316456	0.000584822	336683
**rs1491872**	11	27792891	0.000298373	0.000448331	336683
**rs2764261**	6	108927842	0.00062702	0.0004398	336683
**rs328900**	7	35020280	-0.000654078	0.000457528	336683
**rs382210**	3	84966018	-0.000969853	0.000447392	336683
**rs6533635**	4	113612872	-0.000116642	0.000441378	336683
**rs2005617**	9	33791164	-3.12E-05	0.000438593	336683
**rs2189464**	7	8633758	0.000299714	0.000493901	336683

SNPs.outcome.salad

SNP	chr	pos	beta.outcome	se.outcome	samplesize.outcome
rs13102393	4	63738189	-0.000343218	0.000426908	336683
rs34186148	17	43854655	-0.000387146	0.00043998	336683
rs6482190	10	22037809	-0.000582652	0.000474961	336683
rs7821179	8	83061099	-0.000210646	0.000589881	336683
rs9427220	1	153761750	-9.4E-06	0.000430382	336683
rs57221424	7	35215670	-0.000561692	0.000456962	336683
rs1052352	16	31195279	-0.00042337	0.000425444	336683
rs12908495	15	97011280	-1.64E-05	0.000497727	336683
rs8130508	21	19049865	-0.000949223	0.00047272	336683
rs7619139	3	25110415	0.00143633	0.000432349	336683
rs2194027	5	87822672	-0.0016146	0.000427997	336683
rs75248709	6	92348945	-0.000502277	0.00104601	336683
rs1890012	13	54933682	-8.68E-05	0.000539851	336683
rs4291983	18	38106410	0.00039248	0.000424949	336683
rs4083969	1	231866480	0.0013508	0.000936486	336683
rs17460017	5	137717681	-0.000553746	0.000538866	336683
rs3095337	6	30737591	-0.000875251	0.000526354	336683
rs62461186	7	77730153	0.000109991	0.000553658	336683
rs10819082	9	128645617	0.000780832	0.000451237	336683
rs790561	8	64618026	0.000429594	0.000465535	336683
rs3129962	6	32379547	0.000157312	0.000533313	336683
